# Research progress on nanomaterials in promoting phage function and application

**DOI:** 10.1128/aem.01244-26

**Published:** 2026-08-10

**Authors:** Yi Jin, Zhu Chen, Yiping Wang, Bingqing Xu, Mengye Ma, Jieting Pan, Xingyu Li, Qun Chen, Xibang Liu, Lijian Ding, Liming Jiang

**Affiliations:** 1 Department of Respiratory and Critical Care Medicine, The Affiliated Yangming Hospital of Ningbo University (Yuyao People’s Hospital), Ningbo University 47862, Ningbo, Zhejiang, China; 2 Ningbo Key Laboratory of Malignant Tumor Immunodiagnosis and Immunotherapy, Health Science Center, Ningbo University 47862, Ningbo, Zhejiang, China; 3 School of Basic Medical Sciences, Health Science Center, Ningbo University 47862, Ningbo, Zhejiang, China; 4 Department of Clinical Laboratory Medicine, Ningbo No.2 Hospital, Wenzhou Medical University 26453, Wenzhou, Zhejiang, China; 5 Department of Clinical Laboratory, The Affiliated People's Hospital of Ningbo University 117973, Ningbo, Zhejiang, China; 6 School of Pharmacy, Health Science Center, Ningbo University 47862, Ningbo, Zhejiang, China; The Pennsylvania State University, University Park, Pennsylvania, USA

**Keywords:** phage, nanomaterial, stability, sensitivity, delivery, antimicrobial

## Abstract

Phages, as viruses capable of specifically infecting and lysing bacteria, demonstrate significant therapeutic potential in addressing the escalating challenge of antibiotic resistance. However, their clinical application faces multiple limitations, including poor *in vivo* stability, rapid clearance by the immune system, narrow host range, induction of bacterial resistance, limited efficacy against biofilms, and intracellular bacteria, which severely constrain therapeutic outcomes. Traditional formulation approaches are insufficient to overcome these challenges, making it crucial to explore novel strategies that enhance phage functionality. In recent years, nanomaterials have emerged as innovative solutions through their unique physicochemical properties, offering new perspectives for optimizing phage therapy. This review systematically summarizes and evaluates the latest research progress on various nanomaterials in enhancing phage stability, detection sensitivity, targeted delivery efficiency, and antibacterial efficacy, aiming to provide theoretical references and practical insights for overcoming existing limitations in phage therapy and developing more effective and reliable phage-nanomaterial combination antimicrobial strategies.

**IMPORTANCE:**

Despite increasing interest in phage-nanomaterial systems, the literature in this emerging field remains highly fragmented. Existing studies have explored various nanomaterials to address distinct limitations of phage therapy. These investigations have largely proceeded in parallel across different material platforms, including polymer nanomaterials, liposomes, metal nanomaterials, magnetic nanomaterials, and others, targeting functional outcomes such as stability, detection sensitivity, targeted delivery, and antibacterial efficacy, among others. A systematic synthesis that consolidates these scattered findings and provides a coherent framework across different enhancement strategies is currently lacking. This review contributes to addressing this gap by categorizing recent advances according to the enhanced phage function, summarizing key strategies and mechanisms reported to date, and offering an integrated reference to guide the rational design of phage-nanomaterial combination strategies.

## INTRODUCTION

Phages are highly specific viruses that infect and kill specific bacteria, discovered in the early 20th century and widely present in nature (1). Their size varies depending on the type, usually between 20 nm and 200 nm, with the largest phage exceeding 800 nm (2). The abundance and diversity of phages complicate their classification. According to their morphology, genomic characteristics, and infection mechanism, phages can be classified into temperate phages and lytic phages.

**Fig 1:**
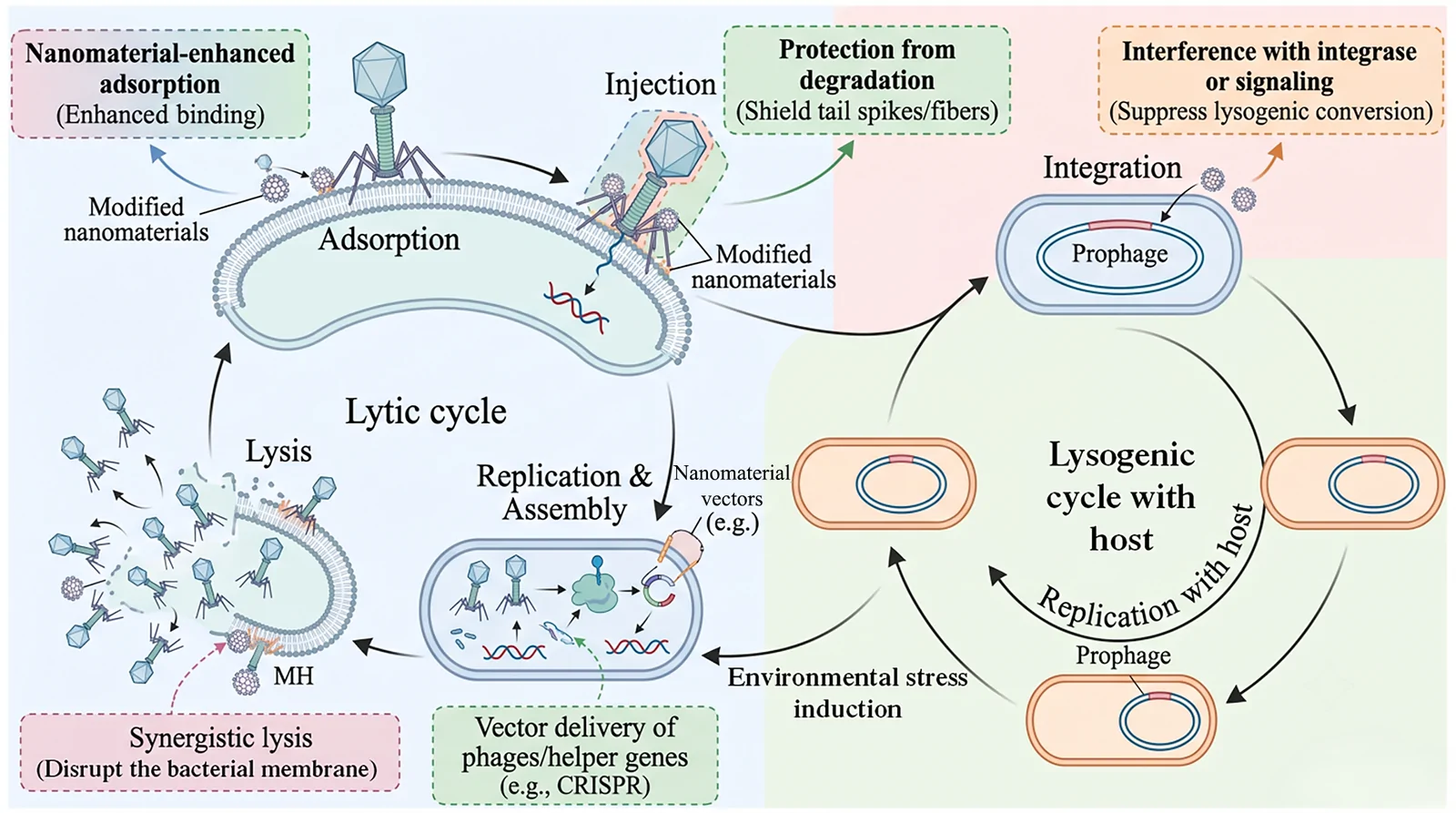
Schematic diagram of phage lytic and lysogenic cycles with potential intervention points by nanomaterials. The lytic cycle includes phage adsorption, injection of genetic material, replication and assembly, and bacterial lysis. The lysogenic cycle involves integration of the phage genome as a prophage, replication with the host chromosome, and subsequent induction to enter the lytic cycle. Colored indicators highlight the steps where nanomaterials have been reported to influence these processes: enhanced adsorption via surface charge modification; protection of phage tail proteins during injection; nanocarrier-delivered genetic cargo (e.g., CRISPR systems or lysin genes); synergistic membrane disruption during lysis (e.g., reactive oxygen species generation); and possible interference with lysogeny (e.g., inhibiting integrase activity or quorum sensing).

Phage life cycles are generally ascribed as being either lytic or lysogenic (Fig. 1) (3). During the lytic cycle, the phage infecting the host bacterium rapidly replicates itself and causes the rupture of the bacterial cell to release the newly produced phage (4, 5). In this process, the hydrolysase (also known as lysozyme) and the membrane protein act together on the bacterial cell wall, leading to cell lysis (5). Conversely, in the lysogenic cycle, the phage genome integrates into the host bacterial chromosome, becoming a temperate phage (6, 7). This integrated state, known as the prophage, can last for a long time, allowing the host bacterium to replicate normally without lysis. Under adverse environmental conditions or when the host bacterium experiences stress, the temperate phage can be reactivated, excise itself from the chromosome, and initiate the lytic cycle, thereby producing new phage particles (7).

As the problem of antibiotic resistance has increased in recent years, infections caused by drug-resistant Gram-negative bacteria have substantially increased the medical burden (8). Furthermore, data from the World Health Organization (WHO) indicate that the mortality rate among patients infected with carbapenem-resistant *Acinetobacter baumannii* is significantly higher than that caused by non-resistant strains (9). Phage therapy is seen as a potential alternative treatment (10). Many studies have demonstrated that phages can effectively target and lyse multiple drug-resistant (MDR) bacteria (11). Notably, these phages specifically infect drug-resistant bacteria without affecting other bacteria or human cells, thus providing a safe and effective therapeutic option. A trial targeting ventilator-associated pneumonia (VAP) showed that targeted phage cocktail therapy resulted in 86% of patients successfully clearing pulmonary infections caused by *Klebsiella pneumoniae* within 14 days, whereas the antibiotic-only group achieved only 57% (12). In another case, Qin and colleagues employed a phage cocktail to treat a man suffering from multifocal urinary tract infections caused by *K. pneumoniae* (13). Similarly, phages (Sa 83 and Sa 87, 1 × 10^5^ PFU) treat chronic sinusitis by killing multidrug-resistant *Staphylococcus aureus* (14). Consequently, phages, by virtue of their unique lytic and lysogeny mechanisms, their ability to precisely target MDR bacteria, and their diverse taxonomic characteristics, represent a pivotal tool in the fight against bacterial infections.

Nevertheless, phage therapy still has limitations in practical applications, with several major limiting factors in terms of application and functionality:

(i) Phages are easily cleared or inactivated by the immune system *in vivo*, resulting in low bioavailability, and consequently, the effect of bacterial infection is poor (15, 16).
(ii) The host range of phages is narrow; therapy may fail if the selected phage does not match the target pathogen or if the infection involves diverse or highly variable bacterial strains (17–19).
(iii) Phage lytic efficiency is reduced by biological limitations, such as lysogenic phenomena, which affects its application (17).
(iv) Analogous to the emergence of antibiotic resistance, the widespread application of phages can select for phage-resistant bacterial variants, thereby diminishing treatment efficacy over time (20).
(v) Phage activity is frequently limited within biofilm structures, rendering phage therapy less effective against biofilm-associated infections (21).
(vi) Phage proliferation depends on bacterial density; improper dosage or timing can cause the phage to be cleared by the immune system before it is fully replicated (22).
(vii) Phages are unable to cross eukaryotic cell membranes, which severely limits their effectiveness against intracellular pathogens, such as *Mycobacterium tuberculosis* and *Salmonella* spp.

To address these inherent limitations of phage therapy, recent research has explored the potential of nanomaterials to expand phage functionality and broaden their therapeutic applications.

Nanomaterials are defined as materials possessing unique physical and chemical properties, distinguished by having at least one dimension in the nanometer range (usually 1 to 100 nm). Due to their minute size and correspondingly large surface area, nanomaterials exhibit significantly different properties from their macroscopic corresponding materials, which gives nanomaterials great potential in electronics, medicine, energy, and other fields (23).

Based on their morphology and dimensionality, nanomaterials can be broadly categorized into one-dimensional (1D) materials, which feature a high aspect ratio with elongated lengths and nanoscale diameters (e.g., nanowires, nanorods, and nanotubes); two-dimensional (2D) materials, possessing a thickness at the nanoscale but extending in two other dimensions (e.g., nanofilms and graphene); and three-dimensional (3D) materials, which are confined to the nanoscale in all three dimensions (e.g., nanoparticles and nanocomposites). There are some specific categories of nanomaterials. For example, peptide nanomaterials can be used for drug delivery and vaccine development due to their excellent biocompatibility and biodegradability, which make them safer and more effective for *in vivo* applications (24, 25). Metallic nanomaterials, such as nano-silver (26) and nano-gold, possess potent antibacterial properties and high electrical conductivity, which have led to their widespread utilization in medicine, electronics, and environmental governance. Furthermore, polyester nanomaterials, such as polylactic acid (PLA) and polycaprolactone (PCL), also have excellent biocompatibility and biodegradability, playing a pivotal role in drug delivery systems and tissue engineering (27).

**Fig 2:**
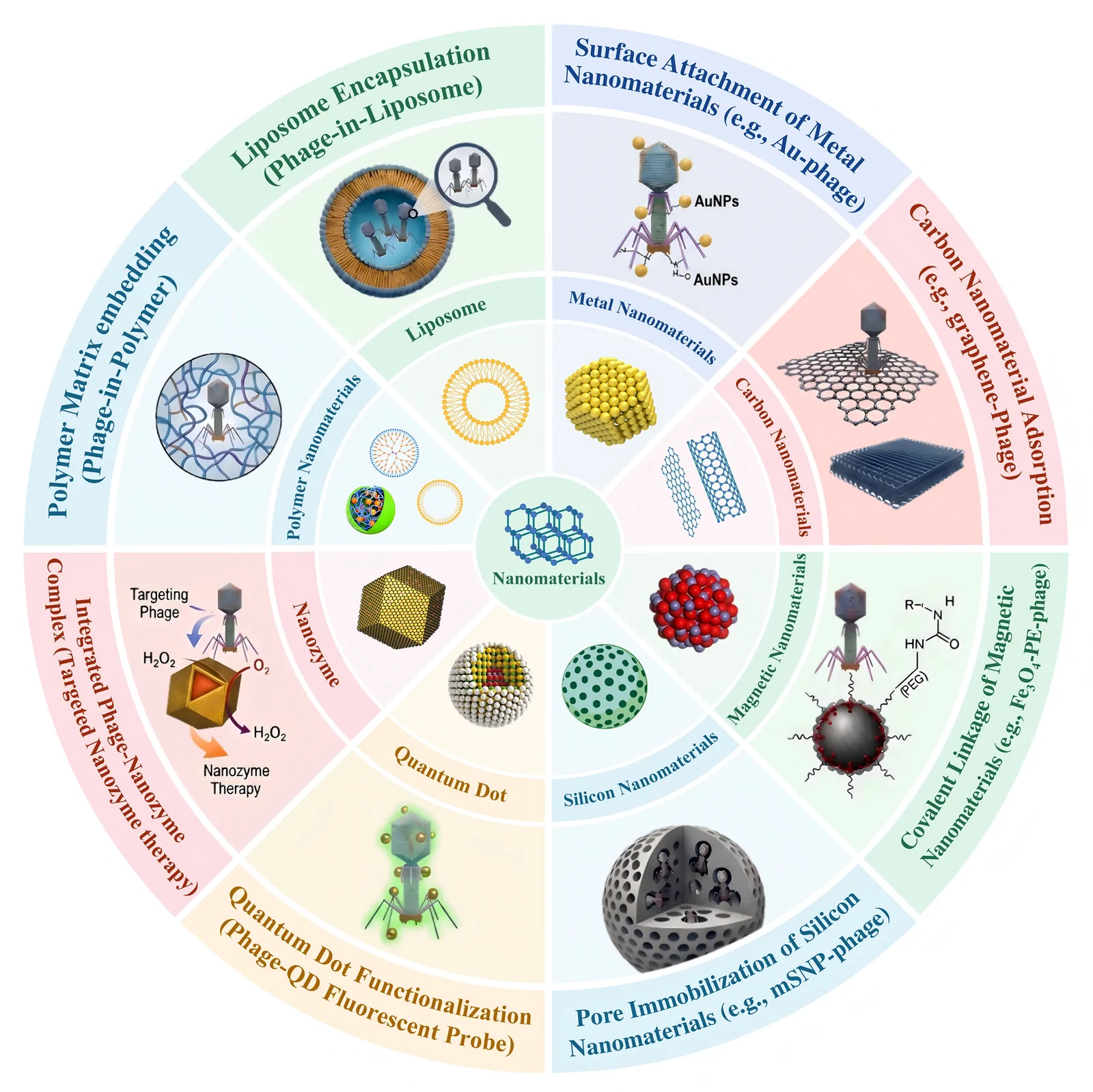
Double-layer schematic diagram of phage-nanomaterial interactions. The figure consists of two concentric rings. The inner ring lists eight nanomaterial classes: liposome, nanozyme, quantum dot, silicon, magnetic, polymer, carbon, and metal. The outer ring shows the typical morphological characteristics of phages after interacting with each nanomaterial. Specifically, metal nanoparticles (e.g., AuNPs) attach to the phage capsid (e.g., Au-phage conjugates); liposomes encapsulate phages within lipid bilayers; carbon nanomaterials (e.g., graphene, MXene) adsorb phages onto their surfaces; nanozymes catalyze reactions (e.g., H_2_O_2_ decomposition) for therapeutic synergy; quantum dots conjugate with phages for fluorescent labeling; silicon nanomaterials (e.g., mesoporous silica nanoparticles, mSNP) immobilize phages within nanopores or on surfaces (e.g., mSNP-phage); magnetic nanomaterials (e.g., Fe_3_O_4_) covalently link to phages; and polymer nanomaterials (e.g., PLGA, chitosan) embed phages in their matrix.

Simultaneously, a growing body of research has demonstrated that the combination of phage therapy and nanotechnology with different types of nanoparticles or microparticles can effectively enhance the function and application effect of phages (Fig. 2). Specifically, certain nanomaterials have been shown to improve the stability, sensitivity, and targeting of phages, as well as enhance phage detection, delivery, and antibacterial ability. In conclusion, we reviewed the progress of nanomaterials in promoting phage function and application.

## NANOMATERIALS PROMOTE PHAGE FUNCTION

Before discussing how nanomaterials promote phage functions, it is helpful to briefly summarize the preparation methods of these composites. Several main approaches have been developed. (i) Physical adsorption relies on electrostatic interactions, hydrophobic effects, or van der Waals forces to attach phages onto pre-formed nanomaterials. This method is simple and avoids chemical damage, but binding is often reversible and susceptible to pH and ionic strength. A notable variation is direct mixing, where the zeta potential of nanoparticles governs phage attachment: values below −35 mV promote tail binding, whereas above +35 mV promote head binding; intermediate values result in non-specific interactions without altering phage performance (28). (ii) Chemical conjugation uses covalent crosslinking between functional groups on phage capsids (e.g., amine and carboxyl) and activated nanomaterials. It provides stable, oriented attachment, yet may reduce phage activity if active sites are affected. (iii) Encapsulation entraps phages within a polymer or lipid matrix (e.g., hydrogels, liposomes, and PLGA nanoparticles) during particle formation. This offers strong protection against enzymatic degradation and enables sustained release, but encapsulation efficiency can vary, and the process may expose phages to organic solvents or shear stress. A related strategy is polymer coating of phage-infected bacteria before lysis, which preserves the intracellular microenvironment and significantly improves phage viability, oral bioavailability, and biofilm penetration; however, it requires precise control of polymer composition and dissolution kinetics. (iv) *In situ* synthesis grows nanoparticles directly onto the phage surface using the phage as a biotemplate, yielding uniform nanostructures while preserving phage recognition. The synthesis conditions (e.g., reducing agents and pH) must be carefully controlled to avoid phage inactivation. An alternative bio-templating approach uses fungal or bacterial cell walls to guide nanoparticle self-assembly into hierarchical porous structures, which can later be loaded with phages for enhanced collective properties (e.g., plasmonic activity), although fine control over deposition remains challenging. The choice of preparation method depends on the intended application (therapeutic delivery, environmental disinfection, or biosensing), balancing simplicity, stability, scalability, and maintenance of phage infectivity.

### Stability

The stability of phages in the external environment is one of the key factors for their application in biomedicine (Table 1). However, the stability of phage is affected by various environmental factors, including temperature and pH. For instance, while phage may remain relatively stable at low temperature, elevated temperature can weaken the interaction between protein molecules. This can lead to denaturation of the phage’s protein structure, thus affecting its structure and function. Vinner et al. have verified that phage titers decrease at 20°C, potentially due to the conversion of urea to ammonia, which causes the virus to inactivate (29). Meanwhile, the stability of the phages is also affected by the environmental pH. In low-pH solution (pH 3.0–4.0), phages are more strongly inactivated than in alkaline solution (pH 11.0–12.0), and phage proteins may denature, leading to a loss of infectivity (30). Phage titers usually decrease slowly with pH. For example, when the pH was reduced from 6.19 to 5.38, *S. aureus* phage titers decreased by 2 log between 4 and 6 h (31);. Conversely, phages were relatively stable under neutral pH conditions (pH 6 to 8) (32). Furthermore, the composition of phages is mainly proteins and nucleic acids, which may be degraded under certain conditions. For instance, phages may show poor stability in pure water, where direct oxidation may lead to the degradation of their capsid and tail structures, ultimately resulting in the loss of genetic material (33). This oxidation effect is more pronounced in high-energy environments and may accelerate the phage degradation process. Alternatively, under extreme environments, the protein coat may lose its normal three-dimensional structure, leading to phage inactivation (30).

**TABLE 1:** Nanomaterials for enhancing phage stability

Nanomaterials	Size	Information	Phage	Function	Reference
Poloxamer 407 hydrogel	Not specified	~18 wt%	IME-AB2 phage	Improved storage stability and enabled sustained release	(34)
Collagen peptide/trehalose-based powder	Not specified	Biocompatible	Not specified	Lyophilization improved survival	(35)
PVA with sorbitol (PVAS20)	Not specified	Film/coating	Not specified	Provide a stable barrier under dry conditions	(36)
Dendrimers	Not specified	Polymeric	General	Enhanced stability via electrostatic or chemical bonding	(37)
Polymeric nanoparticles	Not specified	Hydrophilic	General	Improved stability by packaging	(37)
Sodium alginate (SA) beads	Not specified	Natural polymer	Phage cocktail	Protected survival in low-pH environments	(38)
Chitosan-SA matrix with additives	Not specified	Composite beads	Phage cocktail	Enhanced stability at ultra-low pH	(38)
Liposomes	Nanoscale	–^*a*^	General	Provide physical protection and prolong retention *in vivo*	(39)
Hydrogels (such as pH-sensitive polymers)	Not specified	pH-responsive	General	Enabled controlled release and protection	(39)
Spherical membranous nanoparticles	Nanoscale	Lipid/polymeric	BCP1-BGL phages	Extended retention time *in vivo* and improved antibacterial performance	(40)
TiC MXene nanofragments	Nanoscale	Metal carbide nanofragments	Not specified	Improved adsorption and long-term stability	(41)
Silver nanoparticles (AgNPs)	Not specified	Metal	T7 phage	Enhanced antibacterial efficacy through conjugation	(42)

^*a*^ –, not applicable.

Nanomaterials can effectively improve the stability of phage. This is achieved either by encapsulating phages within a nanomaterial matrix to preserve their structural integrity or by forming a protective barrier that shields them from enzymatic degradation, thereby ensuring their *in vivo* activity. As an example, polymeric nanomaterials can encapsulate phages to protect them from detrimental acidic or alkaline conditions (43). This protective capacity stems from the intrinsic properties of the polymers, which enable the formation of a barrier that prevents phage degradation or inactivation during storage (44). For instance, a Poloxamer 407 hydrogel was shown to have improved storage stability and enabled sustained release of the IME-AB2 phage (34). Similarly, a collagen peptide/trehalose-based powder was found to have improved phage survival after lyophilization (35), and a PVA-sorbitol film provided a stable barrier under dry conditions and achieved a strong release effect (36). Specifically, dendrimers can stabilize phages through electrostatic interactions or chemical bonding to improve their activity during storage (37). This approach was demonstrated to have enhanced phage stability (37). Concurrently, certain polymers can also form stable micelles that enclose phages to provide a protective barrier from their degradation, thereby improving phage stability (45). Consistent with this, polymeric nanoparticles were reported to have improved stability by packaging (37). Meanwhile, polymer nanomaterials can also potentiate antibacterial efficacy through the controlled release of encapsulated phages. Phage titers were markedly reduced when phages incorporated into alginate, alginate-chitosan, alginate-carrageenan, or alginate-whey protein particles were exposed to simulated gastric fluid (46). Related studies concluded that, with the exception of alginate-chitosan particles in which phages were undetectable after only 1 h, all other encapsulation strategies were able to protect the phages from the acidic media (pH 2.5) for at least 2 h (47). Specifically, sodium alginate beads were found to have protected phage survival in low-pH environments, maintaining activity at pH 2.5 (38). Furthermore, a composite chitosan-SA matrix with additives (honey, casein, and gelatin) enhanced stability at ultra-low pH (1.5), offering superior protection compared to pure alginate beads (38). Moreover, pH-responsive hydrogels enabled controlled release and protection, allowing for phage release at specific pH levels while providing a protective barrier against hydrolysis (39).

**Fig 3:**
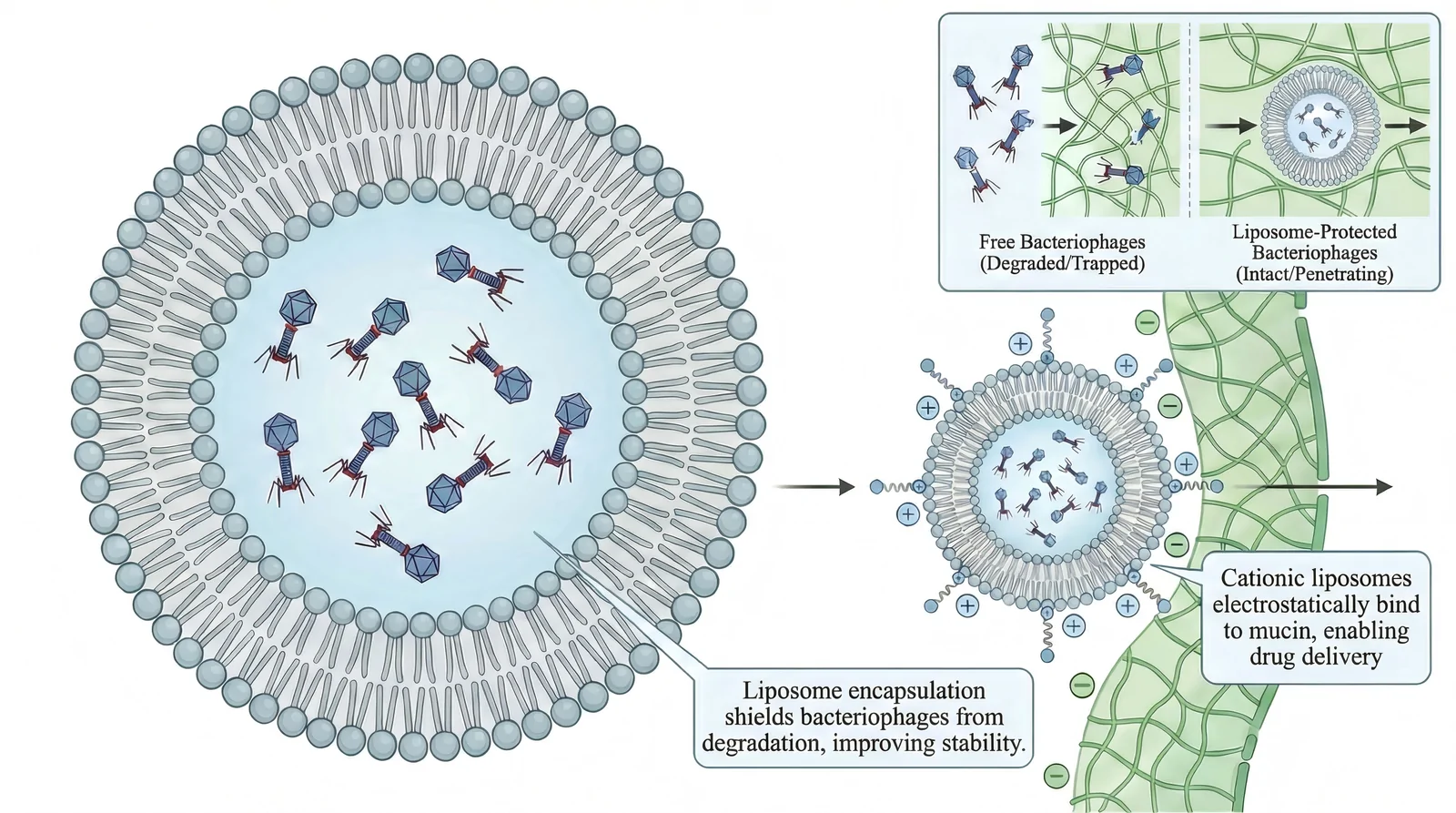
Schematic diagram of liposomes increases the stability and delivery ability of phages by encapsulating them. Free bacteriophages are susceptible to degradation and may become trapped in mucosal barriers. Liposome-protected bacteriophages are protected from enzymatic degradation, remain intact, and can penetrate mucosal layers. Cationic liposomes electrostatically bind to mucin, facilitating drug delivery. This figure illustrates that liposomes, as a single nanomaterial class, simultaneously improve phage stability and *in vivo* delivery efficiency. These two functions are discussed in “Stability” and “Delivery capability,” respectively.

In addition, liposomes are nanostructures composed of phospholipid bilayers, which are widely used in drug delivery systems. Owing to their excellent biocompatibility and low immunogenicity, liposomes are particularly suitable for *in vivo* applications (37). They are able to effectively encapsulate phages, providing physical protection that enhances their stability and prolongs their retention within the body. This capacity of liposomes to provide physical protection and prolong *in vivo* retention has been well documented (39). This protective capacity was demonstrated in a mouse burn model, where five liposome-encapsulated *Klebsiella* phages exhibited extended retention times compared to their free counterparts (48). Similarly, in a mouse *S. aureus* diabetic wound model, using two myoviruses encapsulated in liposomes also had longer phage retention times, with a reported shorter healing time of 33% (49). It had also been observed that, in chickens, one mucovirus and two podoviridae were protected from enzymatic degradation when wrapped in cationic lipid particles, while three *Salmonella* phages were more stable in gastric fluid, prolonging their residence time in animals (50). Notably, liposomes have also been shown to improve the *in vivo* delivery efficiency of phages, a point that will be elaborated upon in the “Delivery capability” section (Fig. 3) (44).

Notably, nanomaterials possess unique physical, chemical, and biological properties. These characteristics enable them to effectively interact with biomolecules such as phages, which can not only enhance the stability of phages but also improve their effectiveness in biological applications through surface modification. From a safety perspective, the long-term biodegradability of these nanomaterials and their potential accumulation *in vivo* require further investigation before clinical translation.

Serving as carriers for drug dissolution, nanoparticles can overcome the continuous biological barriers *in vivo* (51). Phage-nanoparticle conjugates were detected in the bloodstream for a period approximately 24 h longer than that observed for free phages (52). Negatively charged gold nanoparticles were observed to extend the storage time of adenovirus, increasing their half-life from about 48 h to 21 days (from 7 days to more than 30 days at room temperature) (53). In a related approach, spherical membranous nanoparticles were shown to have extended the *in vivo* retention time of BCP1-BGL phages in blood and improved their antibacterial performance in prevention and treatment models (40). Similarly, TiC MXene nanofragments improved phage adsorption and long-term stability in aquatic environments by providing physical protection during extended culture (41), and silver nanoparticles (AgNPs) conjugated with T7 phage enhanced antibacterial efficacy through conjugation, particularly boosting biofilm eradication while maintaining phage stability (42). In a separate context involving vaccine formulation, aluminum oxide nanolayers are used to stabilize the λ phage, thereby ensuring its activity during storage (44). This approach significantly enhanced phage stability under various conditions, particularly across different temperature and humidity conditions.

Among the various stabilization strategies, polymeric nanomaterials (e.g., hydrogels and alginate beads) offer excellent protection against acidic and enzymatic degradation but may suffer from burst release and limited loading capacity. Liposomes provide superior biocompatibility and extended *in vivo* retention but face storage stability challenges. Metallic nanoparticles (e.g., AgNPs and TiC MXene) can simultaneously enhance stability and antibacterial activity, yet their long-term biosafety requires careful evaluation. The choice of nanomaterial should therefore be guided by the specific application context (e.g., oral delivery favors polymer encapsulation, while systemic administration may benefit from liposome formulation).

### Sensitivity and detection capability

Nanomaterials also play a crucial role in enhancing the sensitivity of phages, especially in biosensing applications, which can enhance the binding ability of phages to their target bacteria by enhancing their affinity and selectivity (Table 2) (54). Consequently, this enhanced sensitivity enables phages to efficiently identify and infect target bacteria even at low concentrations. Of note, the multifunctional design of nanomaterials (such as through surface functionalization) can also improve their responsiveness to environmental fluctuations, allowing phages to maintain efficient detection and therapeutic performance in complex biological environments (55, 56).

**TABLE 2:** Nanomaterials for enhancing phage sensitivity and detection capability

Category	Nanomaterials	Size	Information	Phage	Function	Reference
Carbon-based	MXene-TiC nanosheets	20 nm	Negative charge	Not specified	Enhanced phage adsorption stability (>90% activity at 48 h)	(41)
	Graphene nanomaterials	Not specified	High conductivity	M13 phage	Improved biosensor sensitivity and response time	(57)
	Multilayer Carbon Nanotubes (MWCNT)	20–30 nm diam.	High surface area	Phage immobilization vector	Achieved LOD of 3.0 CFU/mL for *E. coli* O157:H7	(58)
	MXene nanosheet	1.1 nm thick	Conductive	Not specified	High-density phage immobilization enhanced electrochemical signal	(59)
Metal-based	Ag/ZnO nanoparticles	Ag: 10–30 nm ZnO: 20–50 nm	–^*a*^	MS2 phage	Enhanced *E. coli* capture rate and reduced detection time	(39)
	Metal nanoparticles (e.g., gold and silver)	Not specified	Plasmonic	Not specified	Formed hybrid probes for visual detection leveraging phage specificity	(60)
	Zinc oxide nanoparticles (ZnO)	30 nm	Photocatalytic	MS2 phage	Increased detection sensitivity by five times	(61)
	Silver nanoparticles (AgNPs)	20 nm	Antibacterial	MS2 phage	Improved bacterial capture rate by 30%, reduced detection time	(61)
	Gold-phage complex	10–15 nm	–	Engineering T7 phage	Enabled visual detection of *B. anthracis* at aM level	(62)
	Magnetic nanoparticles (MNPs)	200 nm	Superparamagnetic	T4 phage	Enabled bacterial detection below 10 CFU in water	(63)
	Vanadium-doped ZnO nanorods	80 nm	Semiconducting	M13 phage	Achieved 12 times higher photocurrent, LOD 3.4 zM for bacterial nucleic acid	(64)
	Bimetallic nanoplasmonic islands (Au/Ag)	Not specified	Solid-state dewetting	Phages with bacterial OMs	Achieved sensitive detection via absorption peak shifts	(65)
	Gold nanoparticles (AuNPs)	15–20 nm	Plasmonic	Phage Simulation Particle	Developed SPR-based detection (1 min, LOD 50 CFU/mL)	(66)
Other	Quantum dots	2–10 nm	Semiconductor; fluorescent	Phage STP55	Served as fluorescent nanoprobe for rapid *Salmonella* detection	(67)
	Surface for phage immobilization	Not specified	–	Not specified	Optimized phage targeting and enhanced bacterial capture efficiency	(68)
	Nluc-labeled phage nanoparticles	Not specified	Luminophore-based	Engineered dual-display phage	Enabled phage bioluminescent immunoassay (P-BLEIA) with low LOD	(69)
	Cu-MOF nanozyme	2–5 nm	Copper-based, polar	PpZDSS02 phage	Enabled chromogenic detection of Proteus penneri (LOD 0.8 CFU/mL)	(70)
	Reporter phages with enzymes	Not specified	–	Virulent phages	Detected trace pathogens and discriminated viable/dead bacteria	(71)

^*a*^ –, not applicable.

Through exploring rapid and sensitive approaches, nanoparticles were confirmed to enhance the functionality of phage-based biosensors (60). Carbon nanomaterials such as graphene and carbon nanotubes have been integrated into phage biosensing platforms. Owing to their high surface area and electrical conductivity, these materials improve sensor sensitivity and reduce response time, rendering them suitable for rapid detection of bacterial pathogens (72). Graphene nanomaterials can enhance the detection of pathogens in electrochemical biosensors by improving sensitivity and response time (57). Metal oxide nanomaterials can improve the sensitivity of fluorescence detection. Notably, zinc oxide nanoparticles synergistically enhance the fluorescence signal of phages, achieving a 5-fold increase in detection sensitivity (61). Furthermore, Zhou et al. demonstrated that exposure of MS2 phages to silver (Ag) and zinc oxide (ZnO) nanoparticles enhanced their capture efficiency against *Escherichia coli* while simultaneously reducing the detection time (39).

Meanwhile, gold nanoparticles are also frequently used due to their unique optical properties and their ability to enhance signal transduction. When conjugated with phages to form “phanorods,” these nanostructures enable the selective targeting and elimination of specific bacterial cells through photothermal ablation. This approach allows local heating, effectively eliminating bacteria while minimizing damage to the surrounding tissue, demonstrating the potential of gold nanorods in phage therapy and detection applications (73). In another study, a nanocomposite consisting of gold nanoparticles, multi-walled carbon nanotubes (MWCNTs), and tungsten oxide nanostructures (WO3) was implemented as a sensor platform. This combination was shown to enhance the electrochemical signals due to the high conductivity and electrocatalytic activity of the nanocomposite, which facilitated the immobilization of phages on the sensor surface. The study reported a limit of detection (LOD) of 3.0 CFU/mL for *E. coli* O157:H7, demonstrating the high sensitivity achieved through this nanocomposite approach (58). Besides, this principle was further illustrated by Mohammed Sedki and colleagues, who physisorbed virions onto a glassy carbon electrode decorated with gold nanoparticles. Following successful incubation, the detection limit was reduced to 14 CFU/mL within 30 min (74). In a further application, Wang et al. specifically and sensitively identified *Bacillus anthracis* using scFv and combined engineered phages with large amounts of gold nanoparticles with gold-binding peptides for signal amplification and visual detection of (75). In a related approach, a gold-phage complex (10–15 nm; gold core-phage capsid) using engineered T7 phage enabled visual detection of *B. anthracis* at the attomolar (aM) level (62). Taken together, electrochemical methods (e.g., using MWCNT/WO3 nanocomposites) achieve detection limits as low as 3.0 CFU/mL but require specialized equipment and longer assay times, whereas optical methods (e.g., SPR-based or visual detection using gold-phage complexes) offer faster readout (within minutes) and attomolar sensitivity, albeit with higher susceptibility to sample turbidity. The choice between these platforms depends on the specific application: high-throughput screening favors optical methods, while point-of-care diagnostics may benefit from electrochemical simplicity.

Magnetic nanoparticles (MNPs) have demonstrated great potential for improving the sensitivity of phage-based detection systems. Specifically, they have been employed for phage immobilization, thereby enabling ultralow detection limits in biosensing applications. A novel method has been developed to genetically engineer phages to produce monomeric streptavidin, enabling efficient and site-specific immobilization on biotin-coated magnetic particles. This approach has been demonstrated by Carmody et al. that the detection limit in water samples was below 10 CFU, thereby underscoring the efficacy of magnetic nanoparticles in enhancing phage-based detection systems (63).

In addition, MXene nanosheets enabled high-density phage immobilization (71 μm²), which enhanced electrode conductivity and improved electrochemical signal detection of *Salmonella* (59). MXene-TiC nanosheets enhanced phage adsorption stability, maintaining over 90% activity within 48 h (41). Parallel to this, vanadium-doped ZnO nanorods combined with M13 phages achieved a 12-fold higher photocurrent signal intensity for bacterial nucleic acid fragment detection, with an LOD of 3.4 zM (64). Silver nanoparticles with MS2 phage improved the bacterial capture rate by 30% and reduced detection time to one-third of conventional methods (61). Bimetallic nanoplasmonic islands (Au/Ag) achieved sensitive detection of phage-bacteria interactions by monitoring absorption peak shifts (65). Furthermore, quantum dots served as a fluorescent nanoprobe with phage STP55 for rapid and efficient detection of *Salmonella* (67). A nanostructured surface for phage immobilization optimized phage orientation and enhanced bacterial capture efficiency, achieving a detection limit of 1 CFU/mL in phosphate-buffered saline (68). These cases demonstrate that numerous experiments have already shown that nanomaterials can effectively enhance the sensitivity and detection capability of phages. Comparing the different platforms, electrochemical sensors (e.g., MXene-based or graphene-based sensors) typically achieve detection limits of 1–100 CFU/mL within 30–60 min, while fluorescence-based methods (e.g., quantum dots or ZnO nanoparticles) offer higher sensitivity (down to single-digit CFU/mL or even zM levels for nucleic acid targets) but often require labeling and may suffer from photobleaching. Photocurrent-based systems (e.g., vanadium-doped ZnO nanorods) provide ultrahigh sensitivity (3.4 zM) but demand sophisticated instrumentation. Thus, the optimal platform should be selected based on the balance between sensitivity, speed, cost, and on-site deployability.

### Delivery capability

As some bacteria are located inside cells, enabling antibiotics or more general anti-infection therapies to penetrate these cells constitutes a major challenge (76, 77). However, phages lack the inherent ability to penetrate eukaryotic cells. To overcome this limitation and facilitate their therapeutic application, nanotechnology-based drug delivery systems have been developed for phage therapy (Table 3).

**TABLE 3:** Nanomaterials for enhancing phage delivery capability

Category	Nanomaterials	Size	Information	Phage	Function	Reference(s)
Lipid-based	Liposomes	Not specified	Lipid bilayer	KPO1K2 phage	Enhanced intracellular delivery against *K. pneumoniae*	(39)
	Cationic liposomes	Not specified	Cationic lipids	KPO1K2 phage	Enhanced intracellular delivery and targeting	(58)
Metal-based	Metal nanoparticle-modified phages	Not specified	Metallic	Generic phage	Improved targeting for intracellular bacteria	(39)
	Fe_3_O_4_ nanoparticles	Not specified	Magnetic	Not specified	Achieved higher phage loading for magnetic targeting	(78)
	Iron-based nanomaterials	Not specified	–^*a*^	Generic phage	Enabled magnetically assisted targeting and controlled delivery	(79)
Polymer-based	Hydrogels	Not specified	Hydrophilic	Generic phage	Provided sustained localized delivery	(39)
	pH-sensitive polymers	Not specified	pH-responsive	fdOVA phage	Triggered release in specific environments for improved targeting	(39)
	Chitosan nanoparticles	Not specified	Cationic, hydrophilic	ΦKAZ14 phage	Protected from enzymatic degradation	(39)
	PLGA	Not specified	Biodegradable, hydrophilic	fdOVA phage	Enabled controlled release for enhanced delivery	(39)
	PLGA	8 μm	Biodegradable polymer	Mixture for *P. aeruginosa*	Improved lung infection delivery	(80, 81)
	Chitosan nanoparticles (CS-NPs)	Not specified	Polymer, cationic	ΦKAZ14 phage	Protected from enzymatic degradation and enhanced delivery viability	(82)
	Nanogel with targeting peptides	Not specified	Peptide-modified	Generic delivery system	Enhances targeting to stroma for co-delivery	(83)
Other	Photosensitizer-modified nanomaterials	Not specified	Photosensitive	Generic phage	Enhanced targeting and penetration in infection niche	(39)
	Engineered phage with peptides	Not specified	Filamentous	M13 phage	Improved cell-specific gene delivery	(84, 85)
	DC-targeting phage	Not specified	Filamentous	DC-targeting phage	Enhanced targeting to dendritic cells and evasion of enzyme degradation	(86)

^*a*^ –, not applicable.

In parallel, novel nanodrug-based delivery strategies have been developed to enhance the internalization of phages (58). Using polymers or lipid-based nanoparticles, phages can be internalized into cells through several endocytic pathways. For instance, Singla et al. developed liposomes loaded with phages to enhance cellular endocytosis and specifically target intracellular *K. pneumoniae* (58). While free phage was able to kill about 20% of intracellular bacteria, loading the phage (KPO1K2) into cationic liposomes increased this killing efficacy to 94.6% (58).

Liposomes also represent a suitable choice for phage delivery due to their biofilm-like properties, which enable them to cross barriers that many phages cannot penetrate on their own. The vesicle size of liposomal nanoformulations, preferably ranging from 50 to 500 nm, can be further optimized through the use of surfactants, various solvents, and lipids, thereby promoting phage delivery into deeper tissue (37). Surface charge represents another critical factor influencing liposome interaction with biological membranes. Positively charged liposomes exhibited greater corneal penetration compared to their neutral and negatively charged counterparts (37). In line with this, cationic liposomes were shown to enhance intracellular delivery and targeting by Singla et al. (58).

This capability stems from the fact that cationic liposomes or positively charged polymers can interact with mucins through electrostatic interactions, thereby enabling controlled drug delivery. For instance, chitosan is a natural cationic polymer, mainly used to adhere to the mucosal layer (87). Gondil et al. encapsulated phage hemolysin into chitosan nanoparticles to treat *S. pneumoniae* infection, demonstrating the mucosal adhesion properties of the particles on the surface of isolated intestinal loops (88). Besides, Nieth et al. developed giant liposomes (average size <5 µm, composed of DOPC, DOPS, and DHPE) for lung delivery (inhalation) and targeting mycobacteria (89). Their experiment demonstrated that liposomes containing phages penetrated cells (monocytes, THP-1 cells) more effectively than free phages (89). While cationic liposomes enhance intracellular delivery, their potential cytotoxicity and the regulatory approval status of such nanoformulations for phage therapy remain important considerations.

In addition, as one type of polymer nanoparticles (PNPs), PLGA exhibits both biocompatibility and biodegradability. It has received FDA approval for various drug delivery systems and has been widely selected for other nanoparticle types (90). Agarwal et al. developed a phage mixture loaded in PLGA beads (8 μm) to treat *P. aeruginosa* lung infections (80). Finally, it was found that phage-loaded microspheres not only killed *P. aeruginosa* within biofilms but also enabled the recovery of high-titer phages from the lungs, and the phages retained the capacity to self-amplify in the presence of their bacterial host. In contrast, free phages exerted no significant effect on bacterial burden (80). Another study confirmed that PLGA enabled controlled release for enhanced delivery of the fdOVA phage (39). Comparing PLGA with liposomal systems, PLGA offers superior aerosolization and sustained release in the lung, whereas liposomes are more effective for intracellular delivery, indicating that carrier choice should be tailored to both the target tissue and the intracellular or extracellular location of the pathogen.

Polymer nanoparticles (PNPs) have also been widely employed to deliver phages for the treatment of ocular infections. These nanoparticles are categorized into two types: nanospheres, in which phages are uniformly dispersed within a polymer matrix, and nanocapsules, where phages are confined to a cavity surrounded by a polymeric membrane. As drug delivery carriers, they have the ability to provide good biocompatibility, improve bioavailability, reduce side effects, and enable targeted tissue delivery (37). Furthermore, PNPs can effectively encapsulate phages and create a protective environment, as exemplified by chitosan-coated alginate (CS-ALG) nanoparticles used for ocular delivery. This formulation has been shown to enhance drug retention in ocular epithelial cells compared with the free drug (91).

In the Ahmad et al. study, chitosan nanoparticles (CS-NPs) and chitosan-ΦKAZ14 phage-loading nanoparticles (C-ΦKAZ14 NP) were prepared and characterized by a simple stress method. Final CS-NPs showed considerable protection of ΦKAZ14 phage against enzymatic degradation, and gel electrophoresis analysis revealed that ΦKAZ14 phages encapsulated within chitosan nanoparticles remained intact, whereas naked ΦKAZ14 phages were completely degraded (82). These findings suggest that chitosan nanoparticles may serve as an effective carrier for the ΦKAZ14 phage and could potentially be used for the oral treatment of *E. coli* infections in poultry (82).

The exploration of nanofibers in enhancing phage delivery capabilities has been a subject of interest in recent studies. One notable experiment involved the use of engineered curli fibers, which were nanofibers produced by the bacterium *E. coli*, to capture and concentrate phages, specifically the MS2 phage. In this experiment, curli-expressing *E. coli* cells were incubated with MS2 virus particles. Subsequently, centrifugation was performed to separate the captured viruses from the supernatant. The results demonstrated a significant increase in the concentration of viable MS2 particles within the curli-cell network compared to negative controls in which curli fiber expression was not induced. Specifically, when NbMS2-functionalized curli fibers were employed, recovery efficiencies of 110% and 119% of MS2 RNA were observed at two different MS2 concentrations (1.6 × 106 and 1.6 × 103 PFU/mL, respectively), indicating the efficacy of these nanofibers in phage concentration (92).

Nanoantibodies are also able to improve the efficiency of gene delivery by fusion with phage genes. Ranjibar et al. fused vascular endothelial growth factor receptor 2 (VEGFR2), a specific nanoantibody, to the capsid gene III of M13 and detected the green fluorescence in 30% of the cells. Approximately 1% of the cells transduced with the recombinant phages were able to express GFP, demonstrating that the M13 filamentous phages were genetically engineered to specifically target mammalian cells for gene delivery (93). Extending this concept, engineered M13 phages with surface peptides (~6.5 nm diameter) were shown to have improved cell-specific gene delivery (84, 85). A DC-targeting phage was also demonstrated to have enhanced dendritic cell targeting (86).

Functionalized magnetic nanoparticles have also been investigated for phage delivery. Through magnetic guidance for phage localization and subsequent release, these nanoparticles typically exhibit high phage-loading capacity and enable efficient phage release under specific conditions. In a related approach, biosurfactant-functionalized Fe₃O₄ nanoparticles achieved higher phage loading for magnetic targeting (78). Besides, iron-based nanomaterials were also reported to have enabled magnetically-assisted targeting (79).

Beyond these strategies, hydrogels were reported to have provided sustained localized delivery for phage therapy (39). pH-sensitive polymers enabled triggered release at acidic sites for improved targeting (39). A nanogel modified with targeting peptides enhanced stromal targeting for co-delivery applications (83). Photosensitizer-modified nanomaterials were shown to have enhanced targeting in infection niches (39).

**Fig 4:**
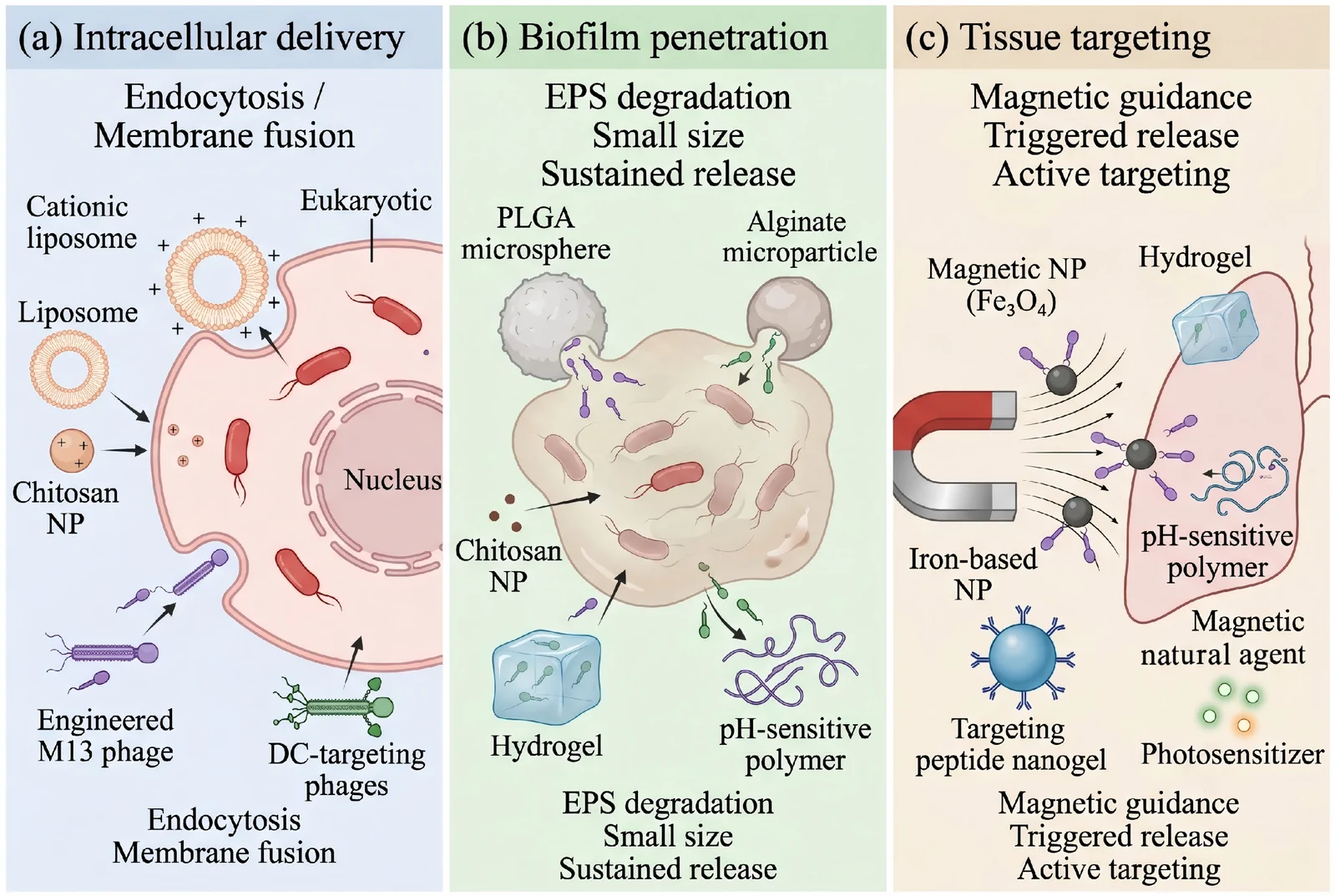
Schematic illustration of nanomaterial-mediated phage delivery for distinct therapeutic scenarios. Three delivery scenarios are shown side by side. (**a**) Intracellular delivery: cationic liposomes (48, 58), liposomes (49), chitosan nanoparticles (70, 82), engineered M13 phage (93), and DC-targeting phages (86) facilitate endocytosis or membrane fusion to transport phages across the eukaryotic membrane, targeting intracellular bacteria. (**b**) Biofilm penetration: hydrogels (34), chitosan NP (97), alginate microparticles (98), and PLGA microspheres (80, 99) provide sustained release and small-size penetration; extracellular polymeric substance (EPS) degradation helps phages reach biofilm-embedded bacteria. (**c**) Tissue targeting: magnetic nanoparticles (Fe_3_O_4_) (78), iron-based nanoparticles (79), targeting peptide nanogels (83), and photosensitizer-modified nanomaterials (100 enable magnetic guidance, triggered release, and active targeting for site-specific phage delivery. This figure highlights that each scenario requires distinct nanomaterial designs based on the underlying mechanistic challenges.

Collectively, these experiments demonstrate that the targeted design of nanomaterials constitutes a significant advantage in phage applications. By enabling targeted recognition of specific bacteria, phages can be delivered directly to infection sites. Alternatively, by exploiting the inherent specificity of phages for bacterial surface antigens, targeted nanocarriers can be designed to minimize damage to surrounding healthy cells. This selectivity enhances therapeutic safety and reduces the incidence of side effects (94–96). A schematic summary of these three delivery scenarios is provided in Fig. 4.

### Antibacterial capability

When selecting nanomaterials to enhance phage antibacterial function, each platform offers distinct advantages and limitations (Table 4). Polymeric nanomaterials (e.g., chitosan, PLGA, and alginate) provide good biocompatibility and tunable release but may have limited loading capacity. Lipid-based systems (liposomes) excel in protecting phages from enzymatic degradation and facilitating cellular uptake, yet they face challenges in long-term storage stability. Metallic nanomaterials (Ag, Au, ZnO, and Fe_3_O_4_) contribute intrinsic antibacterial activity via reactive oxygen species generation and ion release, creating synergistic effects with phages; however, their potential cytotoxicity requires careful evaluation. Magnetic nanoparticles enable magnetically guided targeting, but their size may hinder deep tissue penetration. Moreover, the potential toxicity of metallic nanoparticles to host tissues, their environmental persistence, and the lack of standardized regulatory frameworks for phage-nanomaterial combinations represent critical safety and translational gaps that warrant systematic evaluation.

**TABLE 4:** Nanomaterials for enhancing phage antibacterial capability

Nanomaterials	Size	Information	Phage	Function	Reference(s)
Silver-palladium nano-cages (AgPd)	50–100 nm	–^*a*^	Not specified	Generated surface-bound ROS to kill bacteria selectively	(42, 101)
Silver nanoparticles (AgNPs)	20–30 nm	Metallic	T7 phage	Synergistic biofilm eradication	(42)
Nanomaterial-phage complexes	Not specified	–	Not specified	Reached 98.3% AMR elimination in wastewater	(78)
Silver nanoparticles (AgNPs)	Not specified	Metallic	ZCSE2 phage	Reduced MIC against *Salmonella*	(102)
SiO₂-Fe₃O₄-TiO₂ nanoparticles	Not specified	Composite metal oxides	T4-like phage	Increased lytic efficiency	(28)
Outer membrane vesicles (OMVs)	Not specified	–	Not specified	Neutralized phages and protected bacteria	(103)
ANPs	Not specified	Phage-mimicking	Not specified	Inhibited and delayed the growth of pathogens	(104)
AgNPs-phage complexes	Not specified	0.1 mg/L	Not specified	Facilitated phage transduction in biofilms	(105)

^*a*^ –, not applicable.

Building on the comparative analysis above, the following sections focus on how different nanomaterials enhance the antibacterial efficacy of phages through synergistic mechanisms.

**Fig 5:**
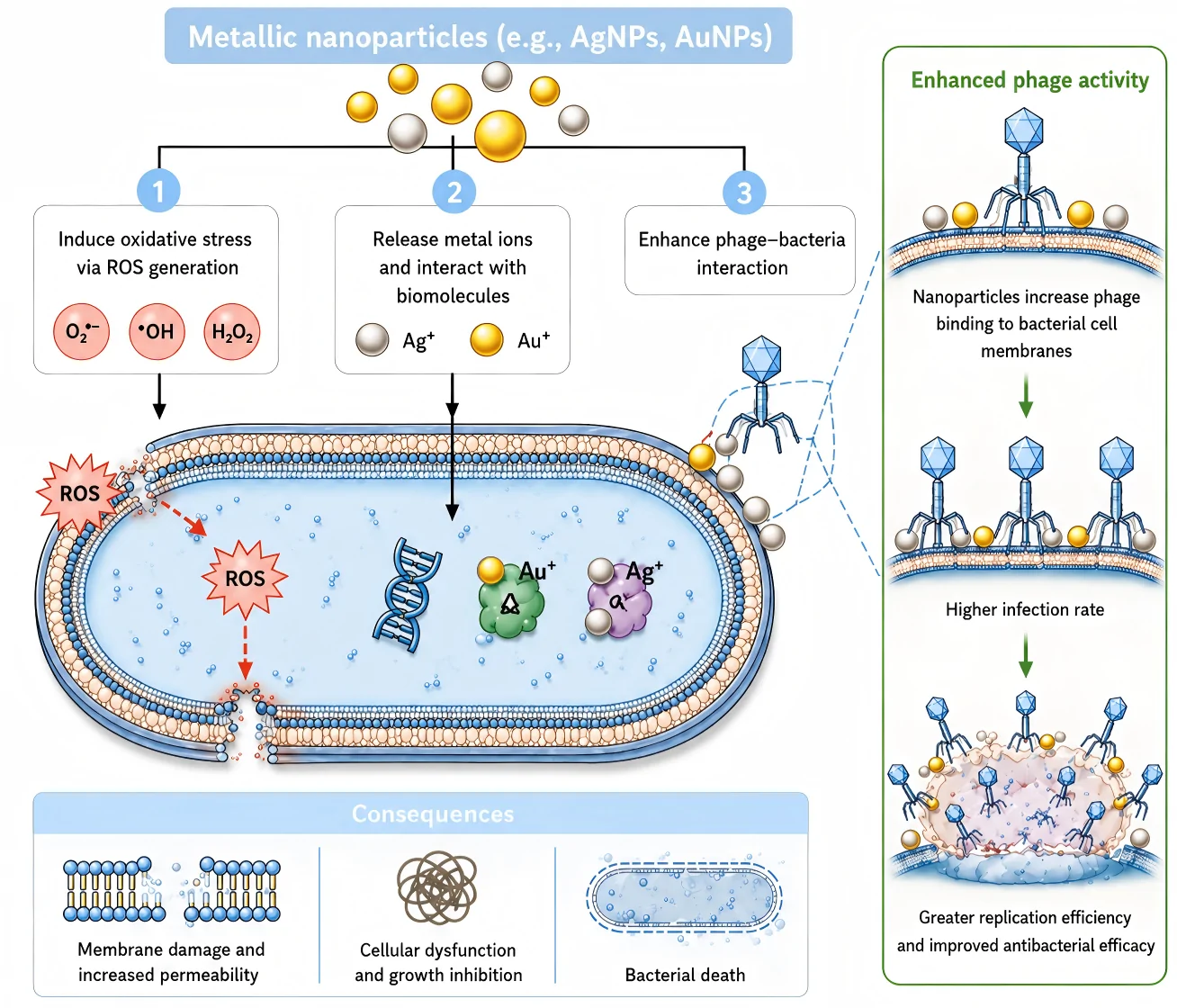
Schematic illustration of the antibacterial mechanisms of metallic nanoparticles in combination with phages. The figure summarizes three key mechanisms: (1) induction of oxidative stress via reactive oxygen species (ROS) generation; (2) release of metal ions (e.g., Ag^+^, Au^+^) that interact with bacterial biomolecules; and (3) enhancement of phage-bacteria interaction, leading to higher infection and replication efficiency. These synergistic effects result in membrane damage, cellular dysfunction, and improved antibacterial efficacy.

Specifically, nanomaterials, particularly metallic nanoparticles such as silver and gold nanoparticles, have demonstrated remarkable efficacy in antimicrobial applications. Their antibacterial mechanisms primarily include: (i) triggering oxidative stress in bacteria by generating reactive oxygen species (ROS), thereby compromising the bacterial cell membrane and inner membrane; (ii) releasing metal ions that subsequently interact with bacterial biomolecules, leading to bacterial dysfunction; and (iii) enhancing the interaction between phages and bacteria. Specifically, the high specific surface area and unique physicochemical properties of nanomaterials enable them to improve the binding capacity of phages to bacterial cell membranes. Consequently, this enhanced binding capacity contributes to increasing both the infection rate and replication efficiency of phages, thereby improving their overall antibacterial efficacy (106). (Fig. 5)

Moreover, the inherent antibacterial properties of phages can be further augmented by nanomaterials. Combining phages with antimicrobial nanomaterials enhances their efficacy against resistant bacteria and helps overcome limitations associated with conventional antibiotic therapy. For instance, T7 phage modified with silver nanoparticles (AgNPs) demonstrated superior efficacy in controlling bacterial biofilms compared to either the phage or AgNPs alone (42). Silver nanoparticles exert a dual antimicrobial mechanism: they directly kill bacteria while simultaneously enhancing the infectious capacity of phages (15, 42). A notable study investigated the combination of AgNPs with ZCSE2, a phage specifically targeting *Salmonella*. The synthesized AgNPs exhibited significant antimicrobial activity, characterized by a distinct color change and enhanced stability upon combination with the phage. Against *Salmonella* at a concentration of 10⁷ CFU/mL, the minimum inhibitory concentration (MIC) and minimum bactericidal concentration (MBC) were determined to be 23 µg/mL (102). The combination of AgNPs at a sub-lethal concentration (0.4× MIC) with the phage showed a notable inhibitory effect, suggesting that the nanoparticles significantly enhanced the antibacterial properties and therapeutic performance of the phage (102). The synergistic effect of biosynthesized silver nanoparticles and phage ZCSE2 has thus emerged as a novel approach against multidrug-resistant *Salmonella* enterica (102).

Notably, in antimicrobial research, synergy is typically defined using quantitative methods such as the fractional inhibitory concentration (FIC) index, where synergy is indicated by an FIC index ≤ 0.5, partial synergy 0.5 < FIC ≤ .75, additivity 0.75 < FIC < 1, neutral 1 ≤ FIC ≤ 4, and antagonism FIC > 4 (107). Other models include the Bliss independence or response surface analysis. When the combined effect merely exceeds the sum of individual components without meeting these criteria, the effect should be considered additive rather than synergistic. In the examples above, the combination of AgNPs and phages showed enhanced killing. However, not all studies have applied formal synergy metrics. Therefore, readers should interpret claims of synergy cautiously, and future studies are encouraged to report standard combination indices.

More importantly, the combined use of multiple nano-metals (e.g., WC, Ag, and Cu) exhibits synergistic antibacterial effects, outperforming individual metals against *S. aureus* and *Pseudomonas aeruginosa* (*P* < 0.01) (108). This synergy arises from different metals targeting distinct bacterial components and pathways, thereby reducing the likelihood of resistance development. Furthermore, combining nano-metals with natural polymers or plant extracts can also enhance antibacterial activity (109). Such multi-metal platforms could potentially be combined with phages to achieve enhanced antimicrobial efficacy, although this integrated strategy remains largely unexplored.

Similarly, another study investigated the interactions between structurally complex, non-filamentous lytic phages and various nanoparticles, including SiO₂, TiO₂, and Fe₃O₄. This research revealed that the zeta potential of nanoparticles played a crucial role in determining phage attachment and, consequently, their lytic performance. Specifically, nanoparticles with a zeta potential below −35 mV facilitated phage tail binding, whereas those above +35 mV promoted head binding. The study concluded that the presence of nanoparticles could modulate phage lytic activity, with the majority of tested nanoparticles exerting a positive effect. Notably, in the presence of SiO₂-Fe₃O₄-TiO₂ nanoparticles, phage lytic efficacy increased by 27% and 45% at 210 and 450 min, respectively, thereby significantly enhancing phage infectivity and overall antibacterial efficacy (28). In contrast, SiO₂ and Fe₃O₄-SiO₂ nanoparticles, which exhibited lower zeta potential values, did not enhance phage activity (28).

Outer membrane vesicles (OMVs) are nanosized proteoliposomes naturally secreted by Gram-negative bacteria that have been shown to transport diverse cargo and interact with phages, thereby influencing phage infection and bacterial survival (110, 111). Several studies have investigated the role of OMVs in enhancing phage sensitivity, particularly in the context of bacterial resistance to phage infection. For instance, research has shown that OMVs produced by pathogenic strains of *Vibrio cholerae* can neutralize virulent phages in a dose- and receptor-dependent manner, thereby reducing phage predation. This finding suggests that OMVs may act as decoys by binding to phages and preventing them from infecting bacterial cells (103). In another study focusing on *P. aeruginosa*, OMVs were found to provide passive protection against phage infection. Specifically, OMVs derived from a hypervesiculating mutant of *P. aeruginosa* neutralized specific phages, indicating that these vesicles not only serve as a physical barrier but also carry receptors that facilitate phage binding. Consequently, this interaction can lead to abortive infections, in which the phage fails to complete its life cycle, thereby enhancing the survival of the bacterial population (111, 112).

Nanozymes, which are nanomaterials with enzyme-like properties, can generate reactive oxygen species (ROS). In the context of phage therapy, the generation of surface-bound ROS by nanozymes enables selective targeting of bacterial cells while minimizing damage to mammalian cells, potentially enhancing phage efficacy without compromising host tissues (101). For example, silver-palladium bimetallic alloy nanocages (AgPd) have been shown to generate surface-bound ROS that selectively kill bacteria over mammalian cells, providing a mechanistic basis for nanozyme-phage synergy (42). These findings suggest that, when combined with phages, nanozymes could potentiate the overall antibacterial effect by creating an unfavorable environment for bacteria. Furthermore, the combination of phages and nanozymes may yield synergistic effects, wherein the phage’s capacity to infect and lyse bacteria is complemented by nanozyme-induced oxidative stress. This dual approach could prove particularly effective against biofilm-forming bacteria, which are notoriously recalcitrant to antibiotic treatment alone. The presence of nanozymes may disrupt biofilm integrity, thereby rendering bacteria more susceptible to phage infection (113).

In certain studies, the integration of phages with nanomaterials has demonstrated promising applications. For instance, in wastewater treatment plants, phages have proven capable of effectively eliminating biofilms formed by antibiotic-resistant bacteria. Another investigation explored the use of phage-mimicking antibacterial nanoparticles (ANPs) designed to structurally resemble phages. These nanoparticles consisted of silver-coated gold nanospheres deposited on a silica core and were engineered to replicate the protein-turret distribution observed on certain phage heads. The study demonstrated that these phage-mimicking ANPs effectively inhibited the growth of various nosocomial pathogens, including *S. aureus* and *P. aeruginosa*, with inhibition rates ranging from 21% to 90%. Importantly, these nanoparticles did not exhibit significant cytotoxic effects on human skin cells, highlighting their potential for safe clinical application (104). In addition to these findings, other nanomaterial-phage combinations have demonstrated unique antimicrobial mechanisms. Nanomaterial-phage complexes (NMCs) with a zeta potential of −22.2 mV eliminated 98.3% of antibiotic-resistant microorganisms in wastewater (78).

While most studies focus on lytic phages for their rapid bactericidal action, temperate phages (which integrate into the host genome as prophages) pose unique risks that may be exacerbated or mitigated by nanomaterials. Key concerns include horizontal gene transfer: temperate phages can carry antibiotic resistance genes or virulence factors from one bacterium to another. For example, AgNPs-phage complexes at a concentration of 0.1 mg/L facilitated phage transduction in *E. coli* biofilms attached to microplastics, directly demonstrating that nanomaterials can promote resistance gene transfer (105). Additionally, lysogenic conversion can alter bacterial phenotypes (e.g., increased biofilm formation or toxin production), potentially reducing the net therapeutic benefit. Moreover, the reduced lytic efficiency of temperate phages compared to strictly lytic phages means that nanomaterial formulations designed to enhance phage stability or delivery may inadvertently prolong the survival of prophage-carrying bacteria. Notably, nanoparticle surface properties (e.g., zeta potential) could influence the lysis-lysogeny decision: certain NPs preferentially bind to phage tails or heads, potentially altering phage structural integrity and shifting the balance toward lysogeny under suboptimal conditions. Regulatory agencies therefore recommend using strictly lytic phages devoid of lysogeny-related genes for therapeutic applications. Accordingly, when designing phage-nanomaterial therapies, careful selection of lytic phages (or genome-engineered phages lacking integrase genes) is essential. Future research should explicitly report whether the phages used are strictly lytic and assess any prophage content, particularly when nanomaterials are employed to enhance phage persistence.

Overall, the synergistic effects observed between nanomaterials and phages across multiple studies suggest that this combinatorial approach can more effectively combat bacterial infections, offering a promising avenue for addressing antibiotic resistance. Further research and clinical trials remain essential to fully realize the therapeutic potential of these innovative strategies.

## ENHANCING THE APPLICATION OF PHAGES IN ANIMALS

The current literature concerning the role of nanomaterials in enhancing phage function and application remains in a developmental phase. Several studies have begun to explore the interactions between phages and various nanomaterials, highlighting their potential applications and safety in different therapeutic settings. Although phage therapy is generally considered safe, the introduction of nanomaterials may complicate this safety. For example, immune recognition of nanoparticles can lead to their rapid clearance from the body, and if phages are also cleared together with the nanoparticles, this may reduce the effectiveness of phage therapy (114). Therefore, many teams have used animal models to evaluate both pharmacokinetic outcomes (e.g., circulation time and tissue retention) and therapeutic efficacy (e.g., bacterial load reduction, survival rate, and wound healing). These animal studies also provide preliminary evidence on the biocompatibility and biodegradability of nanocarriers, although regulatory approval for human use will require more comprehensive safety data. Besides, direct cross-study comparison of these quantitative outcomes is complicated by substantial differences in animal models, infection sites, phage types, nanomaterial characteristics, and dosing protocols. Therefore, reported values should be interpreted as context-dependent rather than as absolute benchmarks.

For instance, Wang et al. demonstrated through *in vivo* mouse studies that, after administration of phage chloroe6 (Ce6)-manganese dioxide nanocomposites (PCMs) and NIR irradiation, the healing rate of wounds infected with *S. aureus* was significantly accelerated, pathogen burden was reduced to negligible levels (~2.5%), and local inflammation was attenuated (100). The PCM-mediated phages within the photodynamic therapy (PDT) framework exerted the predominant bactericidal effect, while the moderate photothermal therapy (PTT) effect facilitated the regeneration and reorganization of nascent collagen in the cambium layer, thereby mitigating scar tissue formation (100). These exciting performances demonstrate the superior potential of the PCM and biological nanoconjugates for synergistic bacterial targeting, eradication, and biofilm clearance, with an efficacy that substantially exceeds the sum of the individual contributions of each PCM component (100).

Poly(lactic-co-glycolic acid) (PLGA) nanoparticles and microparticles are primarily utilized in pharmaceutical applications. Notably, PLGA has received FDA approval for intravenous administration. Relevant experiments have reported the use of 10 μm PLGA microspheres capable of encapsulating phages targeting *S. aureus* or *P. aeruginosa* (99). Agarwal et al. also successfully developed a phage mixture containing 8 μm PLGA microlets for the treatment of *P. aeruginosa* lung infections (80). *In vitro* assays confirmed that *P. aeruginosa* was effectively eradicated by the phage-loaded microspheres. Concurrently, these microspheres were aerosolized and administered to a murine model *in vivo*. The results revealed that, compared to free phage administration, significantly higher phage titers were recovered from the murine lungs. Moreover, the phages retained their capacity for self-amplification in the presence of their bacterial host. In contrast to liposome-based formulations that require intravenous or intraperitoneal administration, PLGA microparticles are amenable to aerosolized delivery, highlighting the importance of selecting nanocarriers compatible with the intended route of administration and target organ.

Alternatively, Chadha et al. administered a cocktail of five phages via intraperitoneal injection to combat *K. pneumoniae* associated with cationic liposomes (48). From a pharmacokinetic perspective, phages loaded within liposomes were detectable in the spleen, liver, and blood for up to 48 h, while free phages were detectable for only 24 h. Regarding therapeutic efficacy, the experiment used a mouse model of burn wound infection caused by *K. pneumoniae* and found that the bacterial count in the blood of mice treated with cationic liposomes was much lower than that of mice treated with free phages (48). Thus, under this specific burn wound model, the liposome formulation provided both prolonged circulation (a pharmacokinetic advantage) and enhanced bacterial clearance (a therapeutic outcome). In a related study, Chhibber et al. encapsulated phages within cationic liposomes approximately 200 nm in diameter for the treatment of methicillin-resistant *S. aureus* (MRSA) wound infections in mice (49). Compared to the group receiving free phages, mice treated with phage-loaded liposomes exhibited significantly elevated phage titers at the wound site (pharmacokinetic advantage), and this was associated with accelerated wound healing (therapeutic outcome). Thus, the improved delivery translated into true bactericidal efficacy.

Beyond murine models, numerous experiments have been conducted using chicken models. For instance, in a chicken model, alginate microparticles incorporating the antacid CaCO₃ and encapsulating a cocktail of three phages active against *Salmonella* exhibited prolonged intestinal residence times compared to free phages (98). In another investigation, chitosan nanoparticles (CS-NPs) functioned as protective barriers for phages targeting MRSA, facilitating the control of multidrug-resistant MRSA in broiler farm settings (97).

Overall, a substantial body of animal experimentation has substantiated the capacity of nanomaterials to potentiate phage function. Importantly, these studies reveal two distinct categories of benefits: pharmacokinetic advantages (prolonged circulation, enhanced tissue retention, and increased local phage concentration) and true therapeutic outcomes (reduction of bacterial load, accelerated wound healing, and improved survival). While pharmacokinetic improvements often contribute to better efficacy, they do not necessarily guarantee it; direct measurement of bacterial killing remains essential. However, as noted in the first paragraph of this section, direct quantitative comparisons across different studies are hindered by substantial variations in animal models, infection sites, phage types, nanomaterial properties, and dosing regimens. Therefore, reported numerical values such as bacterial load reduction, half-life extension, and healing time should be interpreted as context-dependent rather than as absolute benchmarks. More importantly, the studies discussed above are primarily directed toward comprehensively elucidating the biological implications of integrating nanomaterials into phage therapy and formulating strategies to ensure clinical efficacy and safety. This includes, for instance, the imperative to minimize immune recognition and potential toxicity through rational design of these nanocarriers. Consequently, further research is warranted to elucidate the intricate interactions between nanomaterials and phages, as well as their potential implications for human health, while clearly distinguishing between delivery-related and bactericidal outcomes.

## SUMMARY AND FUTURE PERSPECTIVES

This review mainly discusses the research progress of nanomaterials to promote phage function and application. Phages, as a highly specific antibacterial tool, have great potential in treating multidrug-resistant bacterial infections. However, phages have some limitations in practical applications, such as poor stability and easy clearance by the host immune system. The integration of nanomaterials provides new ideas for solving these problems.

The exploration of nanomaterials to enhance phage function represents an emerging field with substantial potential; nonetheless, it confronts several notable challenges and obstacles. A primary hurdle resides in the inherent complexity of nanoparticle chemistries and their interactions with biological systems. The interplay between nanoparticles and immune cells, particularly macrophages, remains incompletely elucidated, and this knowledge gap directly affects the *in vivo* clearance of phage-nanomaterial complexes. Macrophage phagocytosis is highly phenotype-dependent: classically activated (M1) macrophages exhibit greater phagocytic capacity than alternatively activated (M2) macrophages (115). Beyond macrophages, complement proteins (e.g., C1q and C3b) can directly bind and neutralize phages independent of antibodies, particularly affecting myophages by blocking tail contraction. Nanomaterials with certain surface chemistries may exacerbate complement activation, leading to opsonization and accelerated clearance. The zeta potential of nanoparticles also influences phage-nanoparticle binding orientation (tail-binding vs head-binding), thereby altering lytic activity and immune recognition (28). Long-term immunogenicity remains largely unexplored. Patients can develop anti-phage antibodies that persist for over one year in some cases, and conjugation with nanomaterials could either mask or expose immunogenic epitopes. Therefore, future studies should systematically evaluate complement activation markers (e.g., C3a and SC5b-9), quantify macrophage uptake across different polarization states, and assess antibody responses over multiple dosing schedules. Addressing these immunological gaps will be essential for the rational design and clinical translation of safe and effective nanomaterial-phage therapeutics.

A further significant challenge pertains to the imperative need for a more profound understanding of the molecular underpinnings governing these interactions. Current knowledge remains insufficient to establish rational design principles for next-generation nanomaterials capable of selectively engaging with phages and bacterial cells, making it difficult to predict how modifications to nanoparticle surfaces will affect their behavior in biological systems, particularly in terms of phage delivery and activity (116). The exploration of nanozymes as a means to enhance the antibacterial efficacy of phages is a burgeoning area of research, especially in the context of antibiotic-resistant bacteria (101). While several studies have provided insights into the underlying mechanisms, specific experiments directly linking nanozymes to improved phage activity have not been extensively documented. The integration of nanozymes with phage therapy thus constitutes a promising avenue for future exploration. It will be crucial to systematically investigate the optimal conditions for their combined application, including nanozyme types, concentrations, and the specific phages employed, as well as to elucidate the interactions at the nano-bio interface (113). The convergence of nanozyme-mediated oxidative capacity with the lytic action of phages may ultimately pave the way for innovative therapeutic modalities targeting antibiotic-resistant pathogens.

Moreover, the evolution of bacterial resistance poses a critical challenge within the context of phage therapy (8–10). As bacteria evolve, they may develop resistance to phages, which can limit the efficacy of phage-nanoparticle combinations. A comprehensive understanding of the dynamics governing phage-bacteria interactions and the potential for resistance development remains essential for devising durable therapeutic strategies. This requires further research into the evolutionary principles that govern these interactions, including the timing of phage application and the diversity of bacterial populations (117).

Additionally, the practical application of phage-nanoparticle systems is also hindered by the need for rigorous safety assessments. The potential for phage mutations, gene recombination, or resistance development must be carefully monitored to ensure that these therapies do not inadvertently lead to new health risks (118). In addition, the integration of nanomaterials into existing therapeutic frameworks presents logistical challenges, including the design of efficient delivery systems that preserve phage stability and biological activity while accounting for nanoparticle physicochemical characteristics, biological membrane interactions, and potential immune responses (116, 119).

Moving from laboratory to clinical products, several translational challenges need to be addressed. These include scalable production methods compatible with good manufacturing practice (GMP), robust quality control for batch-to-batch consistency, storage and transportation conditions that maintain phage viability without disrupting the cold chain, and the establishment of quantifiable design principles linking nanomaterial properties to phage protection and release kinetics. Three concrete research directions are particularly needed: (i) standardized *in vivo* models (e.g., immunocompetent animals with clinically relevant infection sites) to enable cross-study comparison of efficacy and safety; (ii) design rules for nanocarrier-phage conjugation linking physicochemical parameters (size, charge, surface chemistry) to phage loading, release kinetics, and infectivity preservation; and (iii) clinical-scale manufacturing pathways (GMP compliance, lyophilization stability, batch consistency) to translate laboratory prototypes into marketable products. Addressing these challenges will accelerate the clinical translation of phage-nanomaterial systems.

Mechanism-based investigations should also be strengthened. Future studies should focus on how nanomaterials influence phage adsorption, infection, gene expression, and the overall regulation of phage-bacterial interactions, as these mechanistic insights are essential for rational design.

Three emerging frontiers, namely engineered phages, artificial intelligence (AI), and nanomaterials, are increasingly interdependent and promise to elevate the field when integrated synergistically.

Engineered phages can actively empower nanocomposite systems. Beyond their independent functions, synthetic biology and CRISPR-Cas systems enable phages to be genetically programmed to display targeting ligands, anchor peptides, or cell-penetrating moieties that promote selective binding to nanocarriers. This capability allows spatially controlled delivery, stimuli-responsive release, and enhanced tissue penetration, properties that nanomaterials alone cannot achieve (120, 121). Conversely, nanomaterials address key phage limitations such as poor stability, rapid immune clearance, and limited tissue accessibility by providing protective carriers and sustained-release platforms. The reciprocal synergy between engineered phages and nanomaterials thus creates a design space in which the biological specificity of phages and the physicochemical versatility of nanomaterials are mutually reinforcing.

AI is accelerating the rational design of phage-nanomaterial systems. Predictive models based on protein-protein interaction features can forecast phage-bacterium pairing with approximately 90% accuracy, greatly reducing empirical screening (122). When extended to phage-nanomaterial combinations, AI can facilitate the systematic screening of compatible material-phage pairs, predict how surface chemistry (e.g., charge, hydrophobicity, and functional groups) affects phage loading, stability, and release kinetics, and guide the structural optimization of nanocarriers for specific delivery scenarios. Machine learning can also help identify design rules that link nanomaterial physicochemical properties to phage protection and therapeutic performance, thereby accelerating formulation development and reducing trial-and-error experimentation. The integration of AI with experimental high-throughput platforms holds particular promise for tailoring phage-nanomaterial systems to specific pathogens and infection sites.

By integrating these three directions into a unified framework, future research can overcome major hurdles such as narrow host range, immune clearance, and poor predictability. For instance, polymeric nanoscale coatings can preserve phage vitality during release from bacterial hosts, maintain tail protein integrity, and improve biofilm clearance (123). This holistic approach will pave the way for next-generation phage therapeutics that are both precisely targeted and functionally robust.

This also reminds us that interdisciplinary collaboration is needed among researchers in various fields, such as materials science, microbiology, and clinical medicine. Such collaborations are crucial for addressing the multifaceted challenges associated with the development of phage-nanoparticle systems. By combining expertise from different domains, researchers can better understand the mechanisms underlying phage-nanoparticle interactions and develop innovative strategies to enhance their therapeutic potential (124).

Simultaneously, beyond antibacterial applications, nanomaterial-assisted phages may also hold potential in virology. Future exploration in this domain could include virus detection (using nanomaterials to enhance phage-based diagnostic sensitivity), virus delivery (engineering targeted systems to transport phages to virus-infected cells), and immunomodulation (modulating phage-mediated effects on antiviral immune responses).

Beyond these emerging frontiers, another critical issue is the limited comparability of quantitative outcomes across different studies. As noted above in “Enhancing the application of phages in animals,” reported numerical values, such as bacterial load reduction, half-life extension, and wound healing time, vary considerably across experimental settings, including animal models, infection sites, phage types, nanomaterial properties, and dosing regimens. This heterogeneity limits direct cross-study comparison and challenges reproducibility. To improve rigor, we encourage future studies to adopt more standardized designs and to provide detailed raw data. Until such standards emerge, quantitative efficacy metrics should be interpreted as context-dependent rather than absolute benchmarks.

The impact of nanomaterials on phage genomic stability remains largely unexplored, representing an important safety consideration. Additionally, the potential risk of co-selection or horizontal transfer of antibiotic resistance genes via phage-nanomaterial complexes has been suggested. For instance, silver nanoparticles have been shown to facilitate phage-mediated transduction of resistance genes in biofilm-associated bacteria (105). Systematic evaluation of these safety aspects is needed before clinical application.

Regarding knowledge gaps in pharmacokinetics, although our review summarizes available data on phage half-life extension and prolonged *in vivo* retention achieved by various nanomaterials (e.g., liposomes, PLGA microspheres, and gold nanoparticles), systematic pharmacokinetic parameters, including area under the curve (AUC), peak plasma concentration, clearance rate, and volume of distribution, are largely unreported for phage-nanomaterial systems in the current literature. This gap hampers rational dosing regimen design and warrants future investigation.

In summary, nanomaterials offer substantial advantages in enhancing phage function and expanding their applications. From improving stability and detection sensitivity to enhancing delivery capacity and antibacterial efficacy, nanomaterials provide broad prospects for phage research and application, but there are also significant challenges. It is expected that with the advancement of science and technology and the development of nanotechnology, it is anticipated that innovative therapies based on nanomaterials-phages will continue to emerge, which will play an increasingly significant role in the fields of antibiotic resistance, as well as in disease diagnosis and treatment. By combining the advantages of nanomaterials with phages, new ideas and methods can be provided to solve current medical challenges.
